# Impact of sleep quality and physical activity on blood pressure variability

**DOI:** 10.1371/journal.pone.0301631

**Published:** 2024-04-16

**Authors:** Adam de Havenon, Guido Falcone, Cyprien Rivier, Lauren Littig, Nils Petersen, Paul de Villele, Shyam Prabhakaran, William T. Kimberly, Eva A. Mistry, Kevin Sheth

**Affiliations:** 1 Department of Neurology, Center for Brain and Mind Health, Yale University School of Medicine, New Haven, CT, United States of America; 2 Withings Inc., Issy-les-Moulineaux, France; 3 Department of Neurology, University of Chicago, Chicago, IL, United States of America; 4 Department of Neurology, Massachusetts General Hospital, Boston, MA, United States of America; 5 Department of Neurology, University of Cincinnati, Cincinnati, OH, United States of America; Warren Alpert Medical School of Brown University: Brown University Warren Alpert Medical School, UNITED STATES

## Abstract

Increased blood pressure variability (BPV) is linked to cardiovascular disease and mortality, yet few modifiable BPV risk factors are known. We aimed to assess the relationship between sleep quality and activity level on longitudinal BPV in a cohort of community-dwelling adults (age ≥18) from 17 countries. Using Withings home measurement devices, we examined sleep quality and physical activity over one year, operationalized as mean daily step count and number of sleep interruptions, both transformed into tertiles. The primary study outcome was high BPV, defined as the top tertile of systolic blood pressure standard deviation. Our cohort comprised 29,375 individuals (mean age = 58.6 years) with 127.8±90.1 mean days of measurements. After adjusting for age, gender, country, body mass index, measurement days, mean blood pressure, and total time in bed, the odds ratio of having high BPV for those in the top tertile of sleep interruptions (poor sleep) was 1.37 (95% CI, 1.28–1.47) and 1.44 (95% CI, 1.35–1.54) for those in the lowest tertile of step count (physically inactive). Combining these exposures revealed a significant excess relative risk of 0.20 (95% CI, 0.04–0.35, p = 0.012), confirming their super-additive effect. Comparing individuals with the worst exposure status (lowest step count and highest sleep interruptions, n = 2,690) to those with the most optimal status (highest step count and lowest sleep interruptions, n = 3,531) yielded an odds ratio of 2.01 (95% CI, 1.80–2.25) for high BPV. Our findings demonstrate that poor sleep quality and physical inactivity are associated with increased BPV both independently and super-additively.

## Introduction

Increased systolic blood pressure variability (BPV) is associated with adverse effects on multiple organ systems [1, 2]. Prior research has demonstrated that patients with increased BPV have a higher risk of all-cause mortality in disease states ranging from coronary artery disease to diabetes [3, 4]. However, no therapeutic intervention has been proven to lower BPV. There is preliminary data that sleep quality and physical activity level influence BPV. Increased BPV has been associated with poor sleep quality, primarily sleep interruptions and obstructive sleep apnea [5–7]. The association between physical activity and lower BPV has also been demonstrated using both short-term (hours) and long-term (days, weeks or months) blood pressure data [8–10]. Prior research has not examined a potential additive or super-additive effect of sleep quality and physical activity on BPV. Using a large multi-national dataset, we hypothesized that better sleep quality and higher levels of physical activity would have a super-additive benefit on BPV.

## Methods

### Cohort

We obtained a dataset from the Withings corporation (Withings, Inc; Issy-les-Moulineaux, France) [11], which makes health and fitness consumer electronics. Their blood pressure device uses conventional arm-cuff methodology and their actigraphy watch measures all physical activity and converts it to a daily step count [12]. The sleep data is collected using a pneumatic mat under the mattress. Its measurement of sleep interruptions has been previously validated against polysomnography [13, 14].

**Fig 1:**
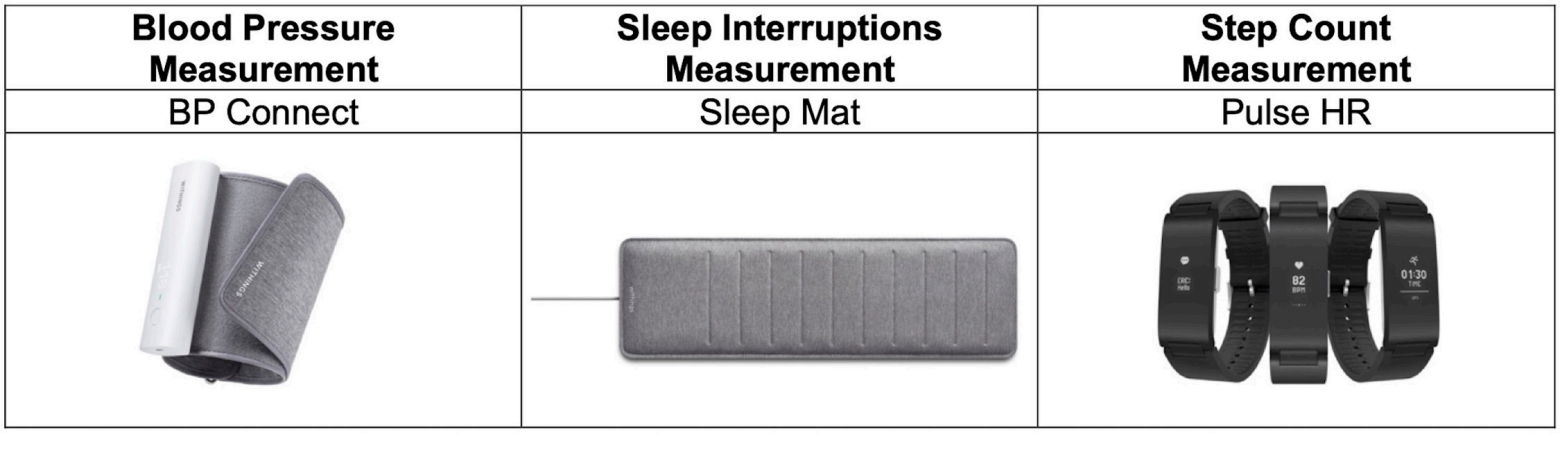
A sample of Withings devices used to acquire physiologic data.

Our cohort comprises community-dwelling adults (age ≥18) who voluntarily recorded blood pressure, sleep, and step count using devices seen in Fig 1. Subjects were not consented for this research but consented to deidentified data sharing when agreeing to Withings’ terms of service. This limited and de-identified dataset was not subject to IRB regulation. We adhered to the STROBE checklist for observational cohort research.

We included individuals who recorded data on all three devices for at least 40 days during the first year after recording started. This inclusion criterion was based on studies showing that the accuracy of BPV measurement stabilizes after 40 blood pressures [15, 16]. We performed two sensitivity analyses in which we included individuals with data on >2 days and >179 days. Systolic blood pressures <50 and >300 mm Hg were considered non-physiologic and converted to missing, as were step counts of 0 or >50,000 and sleep interruptions of >20. Overall, this led to less than 1% loss of data.

### Study outcome, exposure, and covariates

The primary study outcome was BPV. We calculated the standard deviation of daily systolic blood pressure to represent BPV, but as a sensitivity analysis we replicated the primary analysis with two additional BPV methodologies: coefficient of variation and absolute real variability. The main study exposures were step count and sleep interruptions, which is a proxy for poor sleep quality.

**Fig 2:**
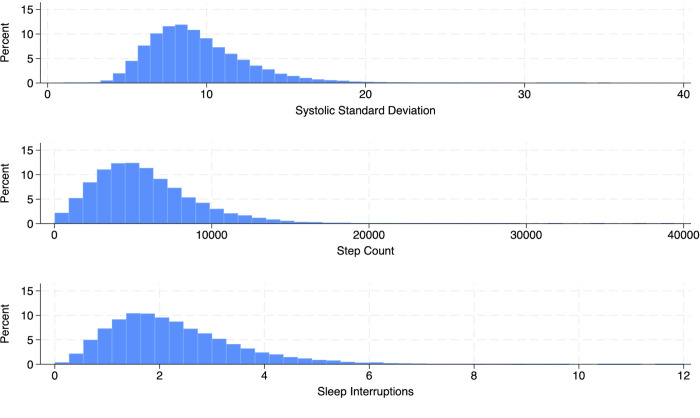
Histograms of the study outcome and exposures.

Withings-derived covariates included country of use, self-reported age, gender and body mass index, which could either be recorded on a Withings scale or calculated using self-reported data. Study derived covariates included total days of measurement, mean systolic blood pressure, and total sleep duration. To improve model fit, we transformed BPV, sleep interruptions, step count, age, and body mass index into tertiles due to right skew (Fig 2), which is common with physiologic and health science data [17–20].

### Statistical methods

We report descriptive statistics for the entire cohort and those with high BPV versus those without. We then fit a logistic regression to the top tertile of standard deviation, which we call “high BPV”. This model’s goodness of fit was confirmed using the Hosmer-Lemeshow test, and the absence of multicollinearity was confirmed by a variance inflation factor of less than 10. We initially fit a linear regression model but, despite transforming the independent and dependent variables, it did not meet the assumptions of linear regression.

After multivariable logistic regression, we report odds ratios and to determine if the exposures were super-additive, we derived the RERI (relative excess risk due to interaction) [21]. We then modeled the exposures as an interaction (steps*interruptions) and used marginal effects to derive the predicted probability of having high BPV for optimal exposure status (highest tertile of step count and lowest of sleep interruptions). We also show the probability of high BPV for a model with continuous values of the exposures in a 3D plot.

## Results

The Withings dataset contained 96,826 individuals, of which 915 were excluded for having no data or non-physiologic data and 66,536 were excluded for not having data on at least 40 days during the year. The remaining 29,375 individuals comprised our primary cohort, which on average had an age of 58.6±12.0 years, measurements on 127.8±90.1 days, systolic blood pressure of 126.5±10.9 mm Hg, daily step count of 5,818±3,370, sleep interruptions of 2.3±1.2, standard deviation of 9.4±3.1, was 82.5% male, and was from 17 different countries. Additional demographics are seen in Table 1.

**Table 1:** Baseline demographics and outcomes in the cohort and after stratification by the highest tertile of BPV versus the others.

Variable	Full cohort (n = 29,375)	Bottom tertiles of BPV (n = 19,584)	Top tertile of BPV (n = 9,791)	P value*
Age	58.6 (12.0)	57.2 (12.0)	61.6 (11.4)	<0.001
Sex				
Man	24,247 (82.5%)	16,712 (85.3%)	7,535 (77.0%)	<0.001
Woman	5,128 (17.5%)	2,872 (14.7%)	2,256 (23.0%)	
Country				
Australia	463 (1.6%)	285 (1.5%)	178 (1.8%)	<0.001
Austria	832 (2.8%)	587 (3.0%)	245 (2.5%)	
Belgium	390 (1.3%)	249 (1.3%)	141 (1.4%)	
Canada	519 (1.8%)	380 (1.9%)	139 (1.4%)	
Finland	416 (1.4%)	307 (1.6%)	109 (1.1%)	
France	3,464 (11.8%)	2,213 (11.3%)	1,251 (12.8%)	
Germany	11,216 (38.2%)	7,363 (37.6%)	3,853 (39.4%)	
Hungary	250 (0.9%)	188 (1.0%)	62 (0.6%)	
Italy	519 (1.8%)	378 (1.9%)	141 (1.4%)	
Japan	1,776 (6.0%)	1,350 (6.9%)	426 (4.4%)	
Netherlands	743 (2.5%)	401 (2.0%)	342 (3.5%)	
Poland	349 (1.2%)	264 (1.3%)	85 (0.9%)	
Spain	388 (1.3%)	280 (1.4%)	108 (1.1%)	
Sweden	325 (1.1%)	221 (1.1%)	104 (1.1%)	
Switzerland	1,063 (3.6%)	741 (3.8%)	322 (3.3%)	
United Kingdom	1,617 (5.5%)	1,094 (5.6%)	523 (5.3%)	
United States	5,045 (17.2%)	3,283 (16.8%)	1,762 (18.0%)	
Body mass index	28.0 (5.2)	27.7 (5.0)	28.6 (5.4)	<0.001
Days of measurement	127.8 (90.1)	131.2 (93.2)	121.0 (83.3)	<0.001
Mean systolic BP	126.5 (11.0)	124.2 (9.9)	131.0 (11.6)	<0.001
Mean systolic SD	9.4 (3.1)	7.7 (1.5)	12.9 (2.6)	<0.001
In-bed time (hours)	8.0 (1.9)	8.0 (1.8)	8.0 (2.0)	0.063
Step count	5818.4 (3370.7)	6141.5 (3464.9)	5172.3 (3073.9)	<0.001
Sleep interruptions	2.3 (1.2)	2.2 (1.2)	2.5 (1.3)	<0.001

*Intergroup differences tested with Student’s t-test for continuous variables and the chi-squared test for binary variables.

Fig 2 shows the distribution of the study’s exposures and outcomes, as well as the right skew that necessitated transforming them into tertiles for accurate modeling. The proportion of individuals in the tertile strata of sleep interruption and step count was well balanced (Table 2), but there is a significantly higher proportion of individuals in the highest tertile of sleep interruptions for those in the lowest (vs. highest) tertile of step count (13.1% vs. 10.1%, p<0.001).

**Table 2:** Distribution of tertiles of activity (step count) and sleep quality (interruptions).

	Lowest tertile of sleep interruptions	Middle tertile of sleep interruptions	Highest tertile of sleep interruptions
**Lowest tertile of step count**	9.2% (2,690)	11.1% (3,263)	13.1% (3,839)
**Middle tertile of step count**	12.1% (3,571)	11.0% (3,235)	10.2% (2,986)
**Highest tertile of step count**	12.0% (3,531)	11.2% (3,294)	10.1% (2,966)

In the adjusted logistic model, the odds ratio of having high BPV for those in the top tertile of sleep interruptions (poor sleep) was 1.37 (95% CI, 1.28–1.47), and for those in the lowest tertile of step count (physically inactive) was 1.44 (95% CI, 1.35–1.54). In the RERI model, the excess relative risk was significant at 0.20 (95% CI, 0.04–0.35, p = 0.012), which confirms that the exposures are super-additive. Comparing those with the optimal exposure status (highest tertile of step count and lowest of sleep interruptions, n = 3,531) to those with the worst exposure status (lowest tertile of step count and highest of sleep interruptions, n = 2,690), the odds ratio for being in the top tertile of BPV was 2.01 (95% CI, 1.80–2.25).

Near identical results were seen for the alternate measures of BPV, which is seen in Table 3. When we include individuals with >2 days of data, the cohort is larger (n = 90,540) and there is a slight reduction in the effect size, but all elements of the analysis remain the same, while in individuals with >179 days of data (n = 7,077) there is an increase in the effect size (Table 4).

**Table 3:** Multiple methodologies to measure high BPV, including standard deviation, coefficient of variation, and absolute real variability.

	Standard deviation	Coefficient of variation	Average real variability
**Top tertile of sleep interruptions**	1.37 (95% CI, 1.28–1.47)	1.38 (95% CI, 1.29–1.47)	1.37 (95% CI, 1.28–1.46)
**Lowest tertile of step count**	1.44 (95% CI, 1.35–1.54)	1.44 (95% CI, 1.35–1.54)	1.43 (95% CI, 1.34–1.53)
**Worst exposure status** **	2.01 (95% CI, 1.80–2.25)	2.01 (95% CI, 1.80–2.23)	1.99 (95% CI, 1.78–2.23)

* Adjusted for age, gender, country, body mass index, days of measurements, mean blood pressure, and total time spent in bed.

** Top tertile of sleep interruptions and lowest tertile of step count, as compared to lowest tertile of sleep interruptions and top tertile of step count.

**Table 4:** Effect of changing inclusion criteria to include more or less individuals based off number of days with available data.

	Sensitivity #1, >2 days of data, n = 90,540	Primary analysis, >39 days of data, n = 29,375	Sensitivity #2, >179 days of data, n = 7,077
**Top tertile of sleep interruptions**	1.27 (95% CI, 1.23–1.32)	1.37 (95% CI, 1.28–1.47)	1.48 (95% CI, 1.29–1.70)
**Lowest tertile of step count**	1.37 (95% CI, 1.32–1.42)	1.44 (95% CI, 1.35–1.54)	1.76 (95% CI, 1.54–2.02)
Worst exposure status**	1.80 (95% CI, 1.69–1.91)	2.01 (95% CI, 1.80–2.25)	2.88 (95% CI, 2.29–3.64)

* Adjusted for age, gender, country, body mass index, days of measurements, mean blood pressure, and total time spent in bed.

** Top tertile of sleep interruptions and lowest tertile of step count.

**Fig 3:**
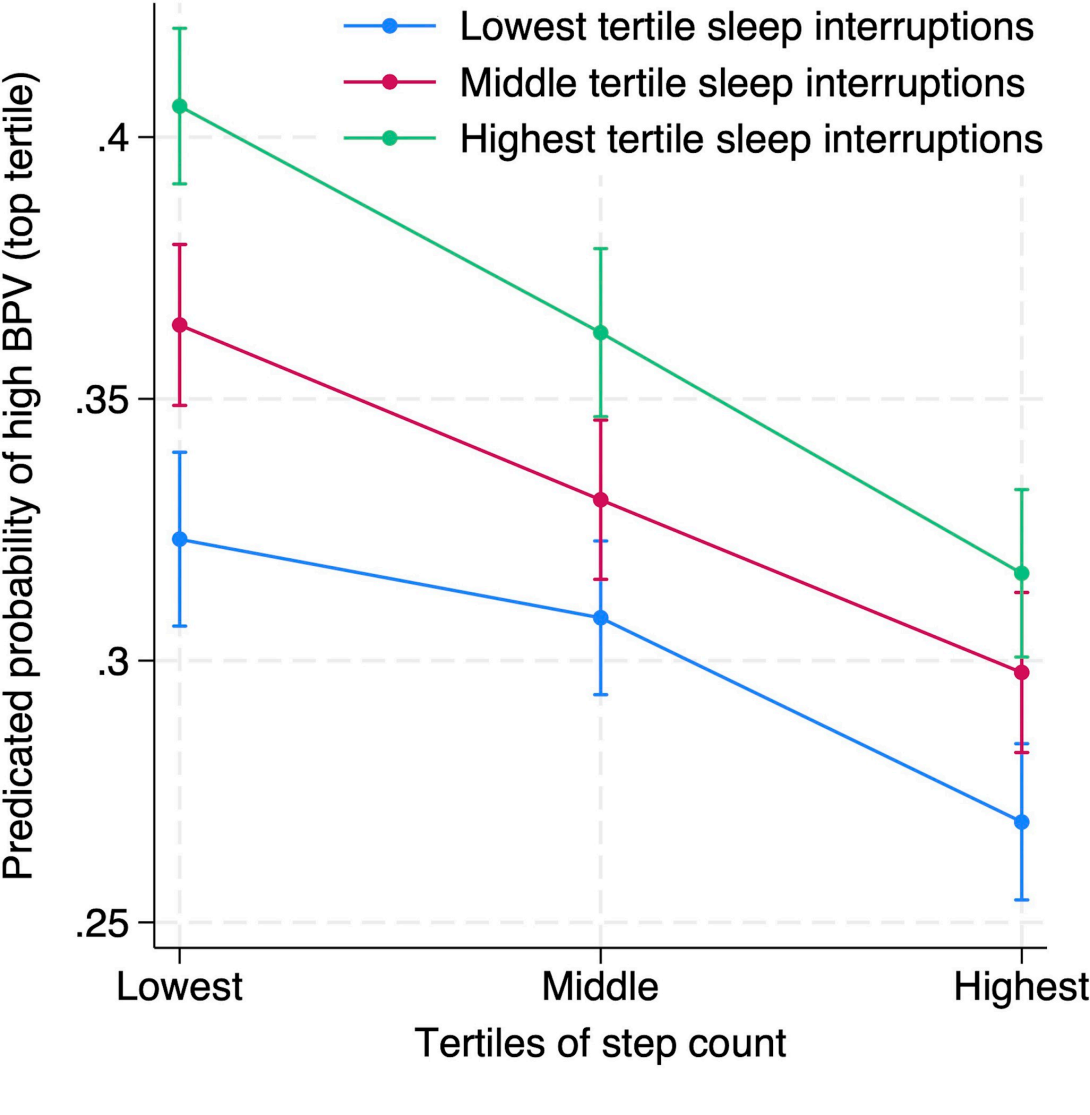
Predicted probability of being in the highest tertile of BPV by tertiles of step count and sleep interruptions. * Adjusted for age, gender, country, body mass index, days of measurements, mean blood pressure, and total time spent in bed.

**Fig 4:**
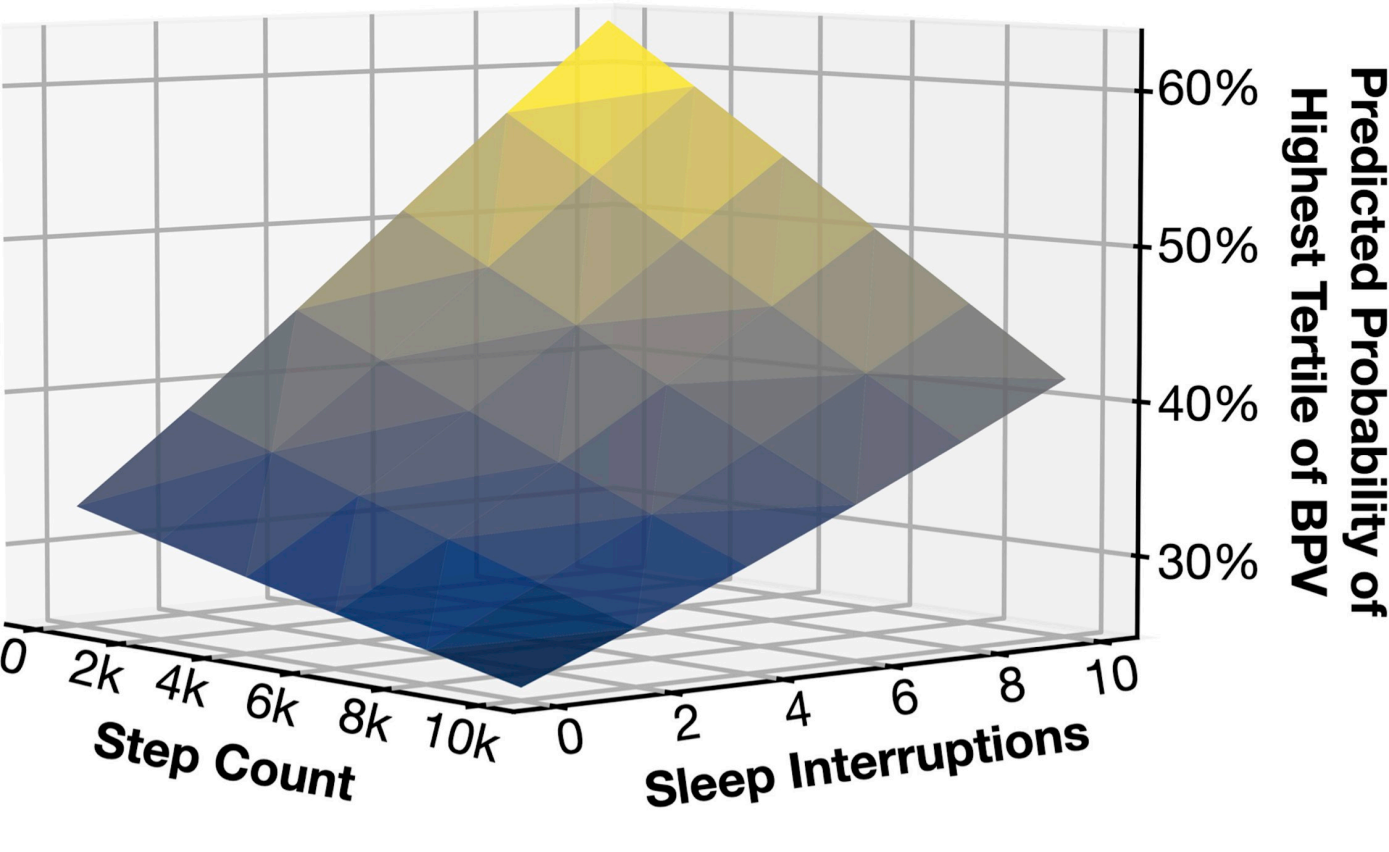
Predicted probability of being in the highest tertile of BPV by continuous values of step count and sleep interruptions. * Adjusted for age, gender, country, body mass index, days of measurements, mean blood pressure, and total time spent in bed.

The predicted probabilities for high BPV are seen in Fig 3. For those with optimal exposure status the probability of having high BPV is 26.9% (95% CI, 25.4–28.4) while for those with the worst exposure status the probability is 40.6% (95% CI, 39.1–42.1) (p<0.001 for difference). The super-additivity is even more apparent when the exposures are treated as continuous variables (Fig 4).

## Discussion

In an international cohort of community-dwelling adults who purchased and used home devices to measure blood pressure, sleep quality, and physical activity, we show that poor sleep quality and physical inactivity are associated with increased BPV both independently and super-additively. This novel finding has not been previously demonstrated because of the lack of datasets with simultaneous home monitoring of blood pressure, sleep quality, and physical activity. Given their novel nature, the findings warrants further study in more generalizable cohorts with additional demographic data and information on medical comorbidities and medications [22]. Despite the possibility of unmeasured confounding, measurement bias, and selection bias in our analysis, the large cohort and frequent and concurrent measurements of the study outcome and exposures are strengths afforded by this unique dataset.

Our findings regarding sleep quality are consistent with prior research that examined 183 patients with obstructive sleep apnea who discontinued treatment and had a subsequent increase in BPV by 1.14 mm Hg [6] and a second study of 3,565 individuals undergoing overnight polysomnogram that found associations between BPV and obstructive sleep apnea severity as well as total sleep time/REM duration after adjusting for sleep apnea severity [5]. Likewise, prior research in small cohorts has demonstrated an association between physical activity and lower BPV, particularly in individuals who are physically fit [8–10]. However, what has been lacking from these prior studies is an examination of the possible synergistic effects of sleep quality and physical activity on BPV. This was an important knowledge gap because prior research has shown an independent connection between activity level and sleep quality [23, 24], which we also showed in our study (Table 2).

A major impediment to developing clinical trials focused on BPV has been that effective treatments to lower BPV are not established. Using a treatment effects simulation of a clinical trial, we previously showed that dihydropyridine calcium channel blockers reduce BPV ∼2 mm Hg [22]. The question of how much BPV would have to be reduced to improve outcomes is not known but it is possible that interventions would have to be combined. Our hypothesis-generating analysis shows that targeting activity and sleep quality may have the potential to lower BPV and could theoretically be used in addition to pharmaceutical approaches.

### Limitations

This study is subject to unmeasured confounding, a limitation that must be recognized due to its potential to result in incorrect interpretations of causal relationships. Unmeasured confounding also compromises the reliability of our findings and their applicability in guiding clinical practice. Consequently, the outcomes of this research should be viewed with caution and not be directly implemented to alter clinical practices or patient care strategies.

We used a convenience sample of primarily male individuals who voluntarily purchased the commercial Withings devices, used them consistently, and sent data to the Withings app. This introduces a selection bias, although the large international sample provides an element of diversity. Nonetheless, the results should not be considered generalizable because disadvantaged socioeconomic groups, older adults, and those with cognitive impairment struggle with implementing mobile health technology [25, 26]. In addition, home devices typically require a Bluetooth connection and internet or cellular transmission of data. More than 25% of Americans over the age of 65 do not have an internet connection [27] and there is a well known digital divide for underrepresented groups [28, 29].

There is also measurement bias. Although home measurement devices offer convenience and the potential for more frequent BP readings, they may differ in accuracy and reliability compared to clinical measurements. We lacked information on medical comorbidities, in particular sleep apnea and cardiovascular diseases. Finally, our study could not account for participants’ medication regimens (e.g. antihypertensive drugs), which could significantly influence the outcome and exposures. Future research should investigate these findings in groups who may not be well represented in convenience samples like this and collect more detailed demographic, medical, socioeconomic, and medication data.

## Conclusion

In summary, our findings demonstrate that poor sleep quality and physical inactivity are associated with increased BPV both independently and super-additively in a convenience sample of primarily male individuals who voluntarily purchased commercial home measurement devices. Given these limitations, our findings need to be replicated in a more generalizable cohort.
